# ﻿Discovery of a new soft-bodied click-beetle genus from Namibia with a unique morphology leads to a modified diagnosis of Drilini (Coleoptera, Elateridae)

**DOI:** 10.3897/zookeys.1213.131283

**Published:** 2024-09-26

**Authors:** Robin Kundrata, Gabriela Packova

**Affiliations:** 1 Department of Zoology, Faculty of Science, Palacky University, 17. listopadu 50, 77146 Olomouc, Czech Republic Palacky University Olomouc Czech Republic

**Keywords:** Afrotropical Realm, Agrypninae, click beetle, distribution, Elateroidea, identification key, male genitalia, new genus, new species, paedomorphosis, taxonomy

## Abstract

Drilini are soft-bodied predatory click beetles with incompletely metamorphosed females. Approximately 150 described species are distributed in the Afrotropical, Palaearctic and Oriental realms, with the highest diversity known from sub-Saharan Africa. In this study, we describe *Namibdrilusalbertalleni***gen. et sp. nov.** from Namibia which brings the total number of genera in Drilini to 16. The discovery of this unique taxon sheds new light on the diversity and evolution of the enigmatic paedomorphic beetle lineage and is interesting for several reasons. This new species is the only known representative of Drilini that has unidentate mandibles and lacks a hook on the dorsal part of the aedeagal median lobe, two of the few characters heretofore used for the unambiguous identification of members of this group. Furthermore, based on its morphology it belongs to a group of genera (*Drilus* clade) which heretofore contained only taxa from the Palaearctic Realm. We provide an updated diagnosis of the tribe Drilini, as well as an updated diagnosis and an identification key for the genera of the *Drilus* clade based on adult males. Further, we explain how to easily recognize adult Drilini from similar-looking soft-bodied elateroids like Elateridae: Omalisinae, Rhagophthalmidae, and Lampyridae: Ototretinae.

## ﻿Introduction

The tribe Drilini (Elateridae, Agrypninae) contains soft-bodied click beetles with flight capable males, incompletely metamorphosed larviform females, and larvae which feed on land snails (Crowson 1972; Baalbergen et al. 2014; Kundrata et al. 2015a; Kundrata and Bocak 2011, 2019). Although the center of diversity of this group lies in the Afrotropical Realm, Drilini are also well represented in the Palaearctic fauna, and several species are also known from the Oriental Realm (Kundrata and Bocak 2019). This group was earlier treated as a separate family, Drilidae, and contained various taxa which are currently placed in several other families within Elateriformia (e.g., Wittmer 1944; Crowson 1972; Geisthardt 2009; Kazantsev 2010). Kundrata and Bocak (2011) placed the group in Elateridae as tribe Drilini in Agrypninae based on a molecular phylogenetic approach. They also transferred genera *Euanoma* Reitter, 1889 and *Pseudeuanoma* Pic, 1901 to Omalisidae (currently a subfamily of Elateridae), and redefined Drilini to contain only *Drilus* Olivier, 1790, *Malacogaster* Bassi, 1834, *Selasia* Laporte, 1838, and tentatively also *Paradrilus* Kiesenwetter, 1866. The latter genus was soon after transferred to Omalisidae by Kundrata et al. (2015b) based on results of a molecular phylogenetic analysis. This concept of Drilini with only three genera lasted only until Kundrata and Bocak (2017) investigated the diversity of Drilini in western Africa and described five new genera from there. Kundrata and Bocak (2019), in the most comprehensive phylogeny of Drilini yet published, defined five main clades, and established six new genera, of which five were from tropical Africa and one from Pakistan. Kovalev et al. (2019) then described a new monotypic genus from Iran.

Here, we report the discovery of a morphologically unique and extremely interesting Drilini specimen from Namibia, which represents a new genus and species. Surprisingly, it is morphologically similar to geographically distant Palaearctic genera. The unique morphology of its mandibles and male genitalia compelled us to modify the diagnosis of the tribe Drilini.

## ﻿Material and methods

The genitalia were dissected after a short treatment in hot 10% KOH. Images of habitus and main diagnostic characters were photographed using a digital camera Canon EOS M6 Mark II attached to a stereoscopic microscope Olympus SZX12. Stacks of photographs were combined with the software Helicon Focus Pro (version 7.6.4, Kharkiv, Ukraine), applying the ‘depth map’ or ‘weighted average’ rendering methods. We did not clean the surface of the holotype in order not to damage the unique specimen. The measurements were taken with a scale bar in an eyepiece. Body length was measured from the fore margin of the head to the apex of elytra (since abdomen is highly flexible in soft-bodied elateroids), body/elytra width at humeri, head width including eyes, minimum interocular distance in the frontal part of the cranium, maximum eye diameter in lateral view, pronotal length at midline, pronotal width at the widest part, scutellar shield length at midline, scutellar shield width at the widest part, aedeagus length medially from base to the apex of the median lobe, and aedeagus width at the widest part. We follow the morphological terminology and the classification of Drilini by Kundrata and Bocak (2019). Label data are cited verbatim. The holotype of the here-described new species is deposited in the National Museum, Prague, Czech Republic (**NMPC**). The Drilini specimens used for comparison of the here-described new species with its congeners are deposited in the following collections: The Natural History Museum, London, United Kingdom (**BMNH**), Koninklijk Museum voor Midden-Afrika, Tervuren, Belgium (**RMCA**), Museum National d’Histoire Naturelle, Paris, France (**MNHN**), Natural History Museum, Budapest, Hungary (**HNHM**), Museo Nacional de Ciencias Naturales, Madrid, Spain (**MNCN**), Naturkundemuseum Erfurt, Germany (**NKME**), Naturhistorisches Museum, Vienna, Austria (**NHMW**), Naturalis Biodiversity Center, Leiden, The Netherlands (**RMNH**), Naturhistorisches Museum, Basel, Switzerland (**NHMB**), Hessisches Landesmuseum, Darmstadt, Germany (**HLMD**), Oxford University Museum of Natural History, Oxford, United Kingdom (**OUMNH**), Museo Civico di Storia Naturale, Genova, Italy (**MSNG**), Museum für Naturkunde, Leibniz-Institut für Evolutions- und Biodiversitätsforschung, Berlin, Germany (**MFNB**), Senckenberg Deutsches Entomologisches Institut, Müncheberg, Germany (**SDEI**), Lund Museum of Zoology, Lund University, Sweden (**MZLU**), Natural History Museum, Copenhagen, Denmark (**NHMD**), and the collections of Albert Allen, Idaho, USA (**PCAA**) and Robin Kundrata, Olomouc, Czech Republic (**PCRK**). The updated diagnosis of the tribe Drilini, as well as an updated diagnosis and an identification key for the genera of the *Drilus* clade, are partly based on Kundrata and Bocak (2019).

## ﻿Systematics

### ﻿Genus Namibdrilus gen. nov.

Taxon classificationAnimaliaColeopteraElateridae

E51A1E16-E0A7-5E26-ACC8-12A3043A6A0D

https://zoobank.org/81EF4D52-F9D3-4BF0-B7FD-8BDBD216032E

Figs 1 , 2 , 3 , 4

#### Type species.

*Namibdrilusalbertalleni* sp. nov.; here designated.

#### Etymology.

The generic name is derived from the Republic of Namibia, plus *Drilus*, a genus name in Elateridae: Drilini. Gender: masculine.

#### Diagnosis.

**Figure 1.**
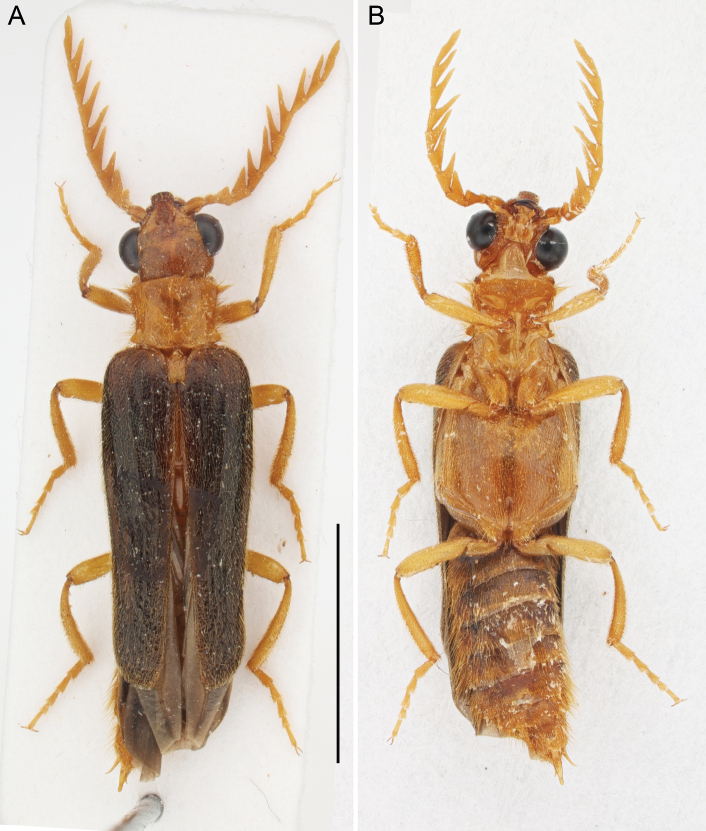
*Namibdrilusalbertalleni* gen. et sp. nov., holotype, male **A** habitus, dorsal view **B** habitus, ventral view. Scale bar: 4.0 mm.

**Figure 2.**
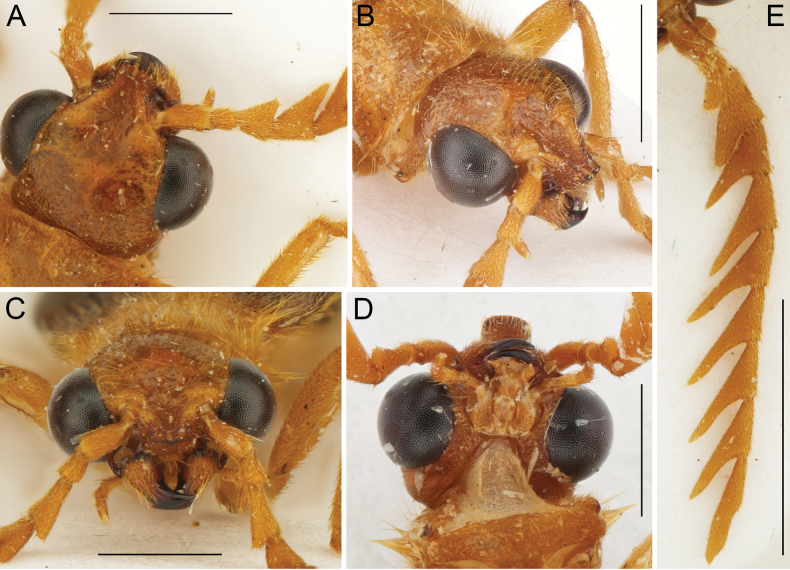
*Namibdrilusalbertalleni* gen. et sp. nov., holotype, male **A** head with basal antennomeres, dorsal view **B** head, frontolateral view **C** head, frontal view **D** head, ventral view **E** left antenna, dorsal view. Scale bars: 1.0 mm (**A–D**); 2.0 mm (**E**).

**Figure 3.**
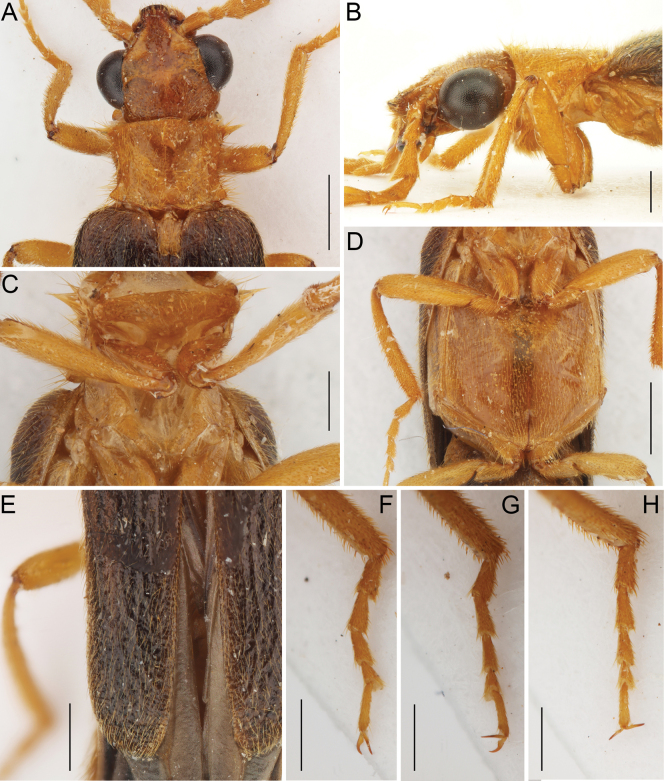
*Namibdrilusalbertalleni* gen. et sp. nov., holotype, male **A** head and prothorax, dorsal view **B** head and prothorax, lateral view **C** prothorax and mesothorax, ventral view **D** metathorax, ventral view **E** apical portion of elytra, dorsal view **F** right protarsus, dorsal view **G** left mesotarsus, dorsal view **H** left metatarsus, dorsal view. Scale bars: 1.0 mm (**A, D**); 0.5 mm (**B, C, E, F–H**).

**Figure 4.**
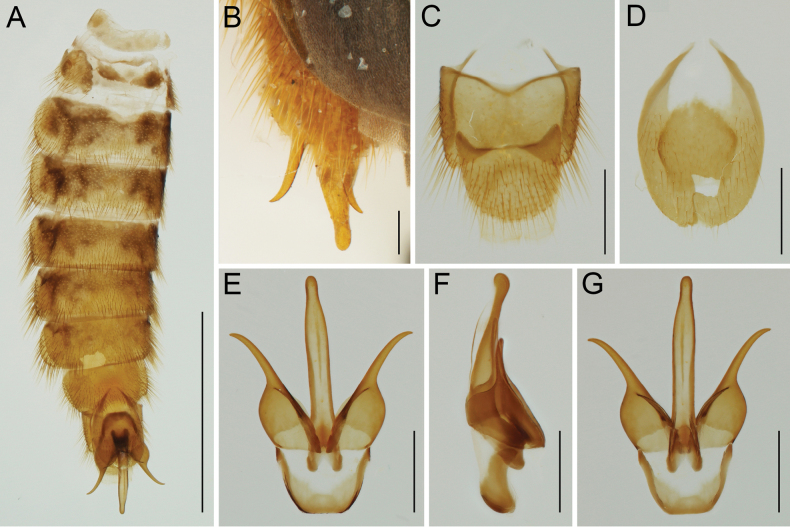
*Namibdrilusalbertalleni* gen. et sp. nov., holotype, male **A** abdomen, ventral view **B** male genitalia (undissected), dorsal view **C** abdominal tergites IX and X, dorsal view **D** abdominal sternite IX, ventral view **E** male genitalia, dorsal view **F** male genitalia, lateral view **G** male genitalia, ventral view. Scale bars: 3.0 mm (**A**); 0.2 mm (**B**); 0.5 mm (**C–G**).

*Namibdrilus* gen. nov. can be unequivocally distinguished from its congeners by the robust unidentate mandibles (Fig. 2A–D) and the aedeagal median lobe dorsally without a subapical hook (Fig. 4B, E–G). Additionally, the following combination of characters can be used to recognize the genus: body (Fig. 1A, B) elongate; frontoclypeal region (Fig. 2A–D) strongly produced forwards, gradually narrowed toward apex, apically carinate and widely rounded; eyes (Fig. 2A–D) large, their frontal separation 1.15 times eye diameter; antenna (Fig. 2E) robust, strongly serrate; pronotum (Fig. 3A, B) roughly subrectangular, 1.30 times as wide as long, lateral carina not developed; prosternum (Fig. 3C) distinctly transverse, without well-developed prosternal process; scutellar shield (Fig. 3A) apically subtruncate and medially emarginate; mesoventrite (Fig. 3C) narrow, v-shaped; elytra (Figs 1A, 3E) elongate, with relatively rough surface and without any apparent striae or rows of punctures; abdomen (Figs 1B, 4A) with eight free visible sternites, the first of which is partly membranous medially; abdominal sternite IX and tergite X (Figs 4C, D) not apparently elongate, about 1.5 times as long as wide. Based on its morphology, *Namibdrilus* gen. nov. is similar to genera of the clade D (*Drilus* clade) defined by Kundrata and Bocak (2019). For more information and the comparison of *Namibdrilus* gen. nov. with presumably related genera see the identification key and discussion below.

#### Distribution.

Namibia.

### ﻿ Namibdrilus albertalleni sp. nov.

Taxon classificationAnimaliaColeopteraElateridae

C9684376-5B08-57F4-8DA9-F39501EDB69F

https://zoobank.org/CFF2DB6F-C808-411D-81B0-323F1B77859F

Figs 1 , 2 , 3 , 4

#### Type material.

***Holotype*** • male, “Namibia, Khomas reg. 140 km SW Windhoek; 23°14.875'S, 16°17.998'E; 1382 m; 14.2.2023; J. Halada lgt.” (NMPC).

#### Etymology.

This species is named after Albert Allen (Idaho, USA), who allowed us to study the unique specimen in his possession, and who kindly donated it to NMPC. For recent discussions about the need for protecting stable biological nomenclatural systems, which also includes the problematics of eponyms, we refer to Jiménez-Mejías et al. (2024).

#### Diagnosis.

As for the genus (vide supra).

#### Description.

**Male. *Body*** (Fig. 1A, B) slightly convex dorsally, 3.30 times longer than width at humeri (8.30 mm long, 2.50 mm wide); yellowish brown to light brown, with head and most of abdomen slightly darker, light brown to brown, and elytra reddish brown to dark brown; body surface covered with yellow pubescence.

***Head*** (Fig. 2A–D) including eyes about 1.15 times as wide as pronotum; surface rather flat, more or less smooth basally, rougher toward apex, punctures sparse and fine but denser and larger basally; frontoclypeal region strongly produced straight forwards between antennae, gradually narrowed toward apex, lateral margins distinctly elevated and thickened above antennal insertions, forming a narrow median furrow, apically again slightly widened, subtruncate, apical margin slightly raised, carinate, very widely rounded. Eyes large, strongly prominent, their minimal frontal separation 1.15 times maximum eye diameter. Labrum covered by frontoclypeal region, visible from frontal view, very short, transverse, with frontal margin slightly concave; sparsely punctate and covered with long semi-erect setae. Mandible unidentate, robust, relatively wide, distinctly curved; base with rough surface covered with long semi-erect setae, apical part shiny. Maxilla with palpus tetramerous, slender, about as long as mandible, palpomere II elongate, palpomere III only slightly longer than wide, terminal palpomere longest, fusiform, apically narrowed, with apex obliquely cut. Labium with palpus trimerous, tiny, less than half length of maxillary palpus, palpomere I short, transverse, terminal palpomere elongate, narrow, fusiform, apically gradually narrowed, obliquely cut. Antenna (Fig. 2E) approximately 4.3 mm long, with 11 antennomeres, robust, pectinate from antennomere 3, reaching humeri when oriented backwards; scapus robust, about 1.8 times as long as wide; pedicel minute, shortest, slightly longer than wide; antennomere 3 about twice as long as pedicel, elongate, with ramus about 0.3 times as long as antennomere itself, antennomere 4 elongate, with ramus about as long as antennomere itself, antennomeres 5–10 subequal in length, elongate, with rami about 1.5–1.6 times as long as respective antennomeres, terminal antennomere twice as long as preceding antennomeres, simple, elongate, about 5.5 times as long as wide, apically narrowed, outer side medially shallowly emarginate.

***Pronotum*** (Fig. 3A, B) roughly subrectangular, 0.6 times as wide as elytra, widest at posterior angles, 1.30 times as wide as long (1.20 mm long, 1.55 mm wide). Anterior margin somewhat widely rounded, slightly produced medially, sides concave, gradually narrowed from anterior margin toward about three-fourths of pronotum length and then distinctly widened at posterior angles, posterior margin simple, trisinuate, rather arcuately and shallowly emarginate medially. Anterior angles inconspicuous; posterior angles short but distinct, divergent, with rough surface, apically narrowly rounded. Lateral carina not developed. Surface of disc relatively rough, sparsely and finely punctate, with moderately dense, long, semi-erect pubescence. Pronotosternal suture very short. Prosternum (Fig. 3C) strongly transverse, its surface uneven, sparsely punctate and covered with semi-erect setae, mainly at frontal margin; prosternal lobe absent, frontal margin almost straight; prosternal process absent. Scutellar shield (Fig. 3A) flat, tongue-like, elongate, approximately 1.70 times as long as wide, with anterior margin gradually declivitous, medially slightly produced, apex subtruncate, medially slightly emarginate. Mesoventrite (Fig. 3C) small, narrow, v-shaped, with posterior margin simply rounded. Mesocoxal cavity open to both mesepimeron and mesanepisternum. Metaventrite (Fig. 3D) large, subtrapezoidal, covered with fine punctures and semi-erect pubescence; discrimen incomplete. Elytra (Figs 1A, 3E) subparallel-sided, only slightly gradually narrowed from humeri to about apical third, both combined 2.30 times as long as wide (5.70 mm long, 2.50 mm wide), 4.75 times as long as pronotum, surface uneven, basally wrinkled, without any distinct striae or lines of punctures, only with several faint costae at basal half, irregularly finely punctate, with long, semi-erect pubescence oriented posteriorly, apices internally slightly divergent, separately rounded; epipleura wider anteriorly, abruptly narrowed near posterior part of metanepisternum, then reduced. Hind wing fully developed. Leg (Figs 1A, B, 3A–D, F–H) moderately long, slightly compressed, with surface covered with moderately long, semi-erect setae, which are thickened mainly ventrally and apically; coxa robust, elongate; trochanter elongate, slightly widened apically, attached obliquely to femur; femur gradually slightly widened towards apex; tibia slightly longer than femur; tarsus (Fig. 3F–H) shorter than tibia, metatarsus relatively longer than pro- and mesotarsus; tarsomeres I–III elongate, widened apically, progressively decreasing in length, tarsomere IV short, ventrally with short membranous lobe, terminal tarsomere long, slender; pretarsal claws simple, slender, slightly curved, basally with long setae.

***Abdomen*** (Figs 1B, 4A) soft, highly flexible, with eight free sternites (II–IX) connected with each other by extensive membranes; sternite II semi-membranous, with two lateral sclerites and two median sclerotizations; all ventrites with sparse, shallow punctures, covered with semi-erect pubescence, which is denser and longer posteriorly and mainly at margins; penultimate ventrite (sternite XIII) with two shallow posterolateral and one rounded median emarginations. Tergites IX and X (Fig. 4C) wider than long, weakly connected by membrane, both covered with fine punctures and relatively long setae; tergite IX basally with two sublateral processes, tergite X apically widely rounded. Sternite IX (Fig. 4D) about 1.5 times as long as wide, roughly oval, with basal portion medially deeply emarginate, apex rounded, surface finely punctate and sparsely covered with setae; sternite X about 0.45 times as long as sternite IX, slightly longer than wide, somewhat rounded. Male genitalia (Fig. 4B, E–G) about 2.6 times as long as wide, about 1.3 times as long as sternite IX; median lobe elongate, distinctly longer than paramere, basally moderately curved in lateral view, with two very short basal struts, dorsally without a subapical hook, rather subparallel-sided in dorsoventral view, slightly widened after half, then slightly but abruptly constricted before apex, apically rounded; paramere distinctly longer than phallobase, with basal half robust and wide, and with apical half distinctly narrowed, divergent, apically narrowly rounded in lateral view; phallobase short, about 1.3 times as wide as long, widely u-shaped.

Females and immature stages unknown.

#### Distribution.

Namibia.

##### ﻿Updated diagnosis of Drilini based on adult males

Body soft, only weakly sclerotized; mandible bidentate (unidentate in *Namibdrilus* gen. nov.); antenna with 11 antennomeres; antennomere II minute, always distinctly shorter than antennomere 3; tarsomere IV shortest, ventrally with short membranous lobe; pretarsal claw with stout setae on outer side of base; abdomen with seven or eight visible sternites (the most basal one can be formed by two separate lateral sclerites connected by membrane); aedeagal median lobe considerably curved laterally, dorsally with subapical hook (without hook in *Namibdrilus* gen. nov.); and phallobase without any posterolateral processes.

##### ﻿Updated diagnosis of clade D (*Drilus* clade) based on adult males

Head often flattened; frontoclypeal region usually more or less produced forwards between antennae; eyes relatively small to medium-sized in Palaearctic species (their frontal separation 1.60–3.00 times eye diameter), large in Afrotropical *Namibdrilus* gen. nov. (their frontal separation 1.15 times eye diameter); antenna serrate to pectinate; pronotum usually less transverse and without sublateral carinae; prosternum without well-developed prosternal process; scutellar shield apically widely rounded to subtruncate; mesoventrite v-shaped, posteriorly simply rounded; elytra often divergent or reduced, with surface uneven, often wrinkled; abdomen with eight visible sternites; sternite IX rounded to oval, basally narrowed; posterior margin of pronotum simple and without emargination or shallowly and arcuately emarginate; abdominal ventrites I–IV never connate.

###### ﻿Genera included

**Figure 5.**
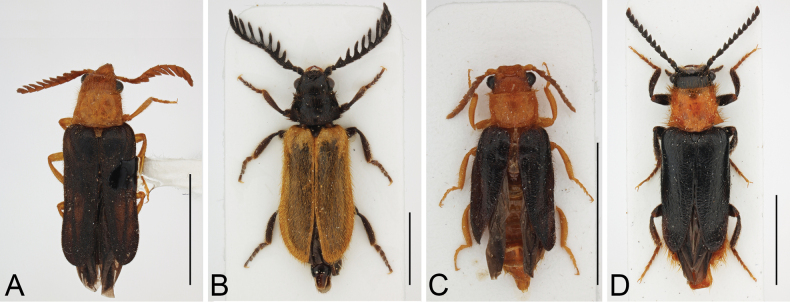
Representatives of Drilini from the clade D (*Drilus* clade), adult males in dorsal view **A***Drilorhinusklimenkoi* Kovalev, Kirejtshuk & Shapovalov, 2019, Iran (PCAA) **B***Drilusflavescens* (Geoffroy, 1785), Italy (PCRK) **C***Malacodrilushajeki* Kundrata & Bocak, 2019, Pakistan (PCRK) **D***Malacogasterpasserinii* Bassi, 1834,Tunisia (PCRK). Scale bars: 4.0 mm.

*Drilorhinus* Kovalev, Kirejtshuk & Shapovalov, 2019 (Fig. 5A); *Drilus* Olivier, 1790 (Fig. 5B); *Malacodrilus* Kundrata & Bocak, 2019 (Fig. 5C); *Malacogaster* Bassi, 1834 (Fig. 5D); and *Namibdrilus* gen. nov. (Figs 1–4). For more information on individual genera see e.g., Kundrata et al. (2015a), Sormova et al. (2018), Kovalev et al. (2019), Kundrata and Bocak (2019) and Hoffmannova and Kundrata (2022).

### ﻿An identification key for the genera of Drilini in clade D (*Drilus* clade) based on adult males

**Table:** 

1	Eyes large, their frontal separation 1.15 times eye diameter; frontoclypeal region strongly produced forwards, anteriorly carinate and widely rounded; mandible unidentate; aedeagal median lobe without any subapical hook; Afrotropical Realm	***Namibdrilus* gen. nov.**
–	Eyes rather small, their frontal separation 1.60–3.00 times eye diameter; frontoclypeal region if produced forwards then anteriorly emarginate; mandible bidentate; aedeagal median lobe dorsally with a subapical hook; Palaearctic Realm	**2**
2	Frontoclypeal region strongly produced forwards and distinctly narrowed apically; mandibles robust, wide and only apically abruptly narrowed; antenna pectinate; Iran	***Drilorhinus* Kovalev et al., 2019**
–	Frontoclypeal region if produced forwards then relatively short and wide; mandibles slenderer, gradually narrowed toward apex; antenna serrate to pectinate	**3**
3	Lateral pronotal carina short, reaching usually no more than half of pronotal length; mandible with only a small tooth medially at incisor; abdominal sternite IX distinctly elongate, about or more than twice as long as wide (except for *M.ruficollis* Dodero, 1925)	***Malacogaster* Bassi, 1834**
–	Lateral pronotal carina almost complete; mandible with distinct tooth medially at incisor; abdominal sternite IX not elongate, always less than twice as long as wide	**4**
4	Frons not distinctly widened; antenna serrate or pectinate; pronotum usually transverse (exceptionally subquadrate); anterior margin of prosternum rounded or straight; first visible abdominal sternite medially membranous	***Drilus* Olivier, 1790**
–	Frons distinctly widened; antenna serrate; pronotum subquadrate; anterior margin of prosternum concave; first visible abdominal sternite complete	***Malacodrilus* Kundrata & Bocak, 2019**

## ﻿Discussion

The here-described new genus of Drilini is extremely interesting from the morphological as well as from the evolutionary point of view. Its discovery has changed our view regarding the diagnosis of the tribe Drilini as well as our understanding of the morphology and distribution of one of the main clades of Drilini. Therefore, both the diagnostic characters used for the recognition of Drilini and the unique morphology of the new genus are worthy of a more detailed discussion.

Within the family Elateridae, Drilini are easily recognizable due to their various modifications connected with the soft-bodiedness and the loss of clicking mechanism. Compared to the typical well-sclerotized and clicking Elateridae, Drilini males have e.g., much softer body, reduced prosternum usually with a strongly reduced or missing prosternal process, reduced mesoventrite without a well-developed mesoventral cavity and often with a reduced mesoventral process, and the abdomen with seven or eight visible sternites (compared to usually five in hard-bodied elaterids; Kundrata and Bocak 2019). The females are larviform, lack elytra, and do not look like any other elaterid adult female (e.g., Hoffmannova and Kundrata 2022).

However, it is not always easy for some to distinguish adult Drilini from similar-looking soft-bodied elateroids, mainly Elateridae: Omalisinae, Rhagophthalmidae, and Lampyridae: Ototretinae, many of which were historically classified in the broadly defined Drilidae (Wittmer 1944). From all three above-mentioned groups, Drilini males were always easily distinguishable based on their bidentate and more robust mandibles, setae at the bases of pretarsal claws (the latter is the character typical for Agrypninae), and a distinct hook on the dorsal part of the aedeagal median lobe. All known Omalisinae, Rhagophthalmidae, and Ototretinae have simple, usually narrow and sickle-shaped mandibles; they lack setae on the pretarsal claws and lack a hook on the aedeagal median lobe (Janisova and Bocakova 2013; Bocek et al. 2018; Kundrata et al. 2022). Furthermore, Omalisinae have antennomeres 2 and 3 short and subequal in size (only antennomere 2 is short in Drilini), and the phallobase with posterolateral processes (without those processes in Drilini) (e.g., Kundrata et al. 2015b, Packova et al. 2024), and Rhagophthalmidae have always 12 antennomeres (compared to 11 in Drilini) and numerous species have deeply emarginate eyes (always simple in Drilini) (Kawashima et al. 2010; Kundrata et al. 2022; Packova and Kundrata 2023). Regarding the females, all these groups have them paedomorphic, retaining many larval characters in their adulthood. Females of many genera and species of Drilini, Omalisinae, Rhagophthalmidae, and Ototretinae are actually unknown. Nevertheless, based on available information, we can recognize females of Drilini by having only the head and legs completely metamorphosed and the rest of the body being larviform (e.g., Kundrata et al. 2015a; for females of Omalisinae see e.g., Geisthardt 1977 and Bocek et al. 2018, for females of Rhagophthalmidae see Kawashima et al. 2010 and Kundrata et al. 2022, and for females of Ototretinae see Kawashima 1999 and Yiu and Jeng 2018).

From three main characters which were heretofore used for the unambiguous identification of Drilini males (i.e., bidentate mandibles, setae at the bases of pretarsal claws, and a distinct hook on the dorsal part of the aedeagal median lobe), *Namibdrilus* gen. nov. has only the setae on the claws. *Namibdrilus* gen. nov. has unidentate mandibles (Fig. 2A–D) that are still more robust than the sickle-shaped mandibles of omalisines, rhagophthalmids and ototretines. However, all other Drilini have bidentate mandibles although some *Drilus* spp. and *Malacogaster* spp. have the inner tooth minute (Kundrata et al. 2015a, Hoffmannova and Kundrata 2022). All Drilini species for which male genitalia was known, representing all described genera (confirmed by several hundreds of dissections by the first author and his students), had an aedeagal median lobe with a distinct hook (see e.g., Geisthardt 2007; Kundrata and Bocak 2017, 2019; Kovalev et al. 2019). However, *Namibdrilus* gen. nov. is surprisingly the first representative of Drilini with the median lobe of the aedeagus simple, without any hook (Fig. 4E–G). Consequently, the diagnosis of Drilini needed to be modified accordingly.

Based on the results of molecular phylogeny supported by the morphological characters, Drilini were divided into five major informal groups, i.e., clades A, W, S, M and D (letters represent the first letters of the respective genus name which is typical for the given clade; Kundrata and Bocak 2019). Four of those clades (A, W, S, M) contain solely or predominantly Afrotropical taxa (with several species of the clade S extending to the Oriental Realm) while the clade D contained exclusively Palaearctic species. One would expect that *Namibdrilus* gen. nov. from southern Africa belongs to one of the African clades; however, surprisingly it falls morphologically into the clade containing Palaearctic species. All known African Drilini differ from *Namibdrilus* gen. nov. in having a short frontoclypeal region and an aedeagal median lobe with a dorsal hook. Furthermore, the representatives of two basal-most clades A (*Austroselasia* Kundrata & Bocak, 2019) and W (*Habeshaselasia* Kundrata & Bocak, 2019, *Latoselasia* Kundrata & Bocak, 2017, *Mashaselasia* Kundrata & Bocak, 2019, *Wittmerselasia* Kundrata & Bocak, 2017) differ from *Namibdrilus* gen. nov. in many important diagnostic characters including the pronotum with posterior margin medially rectangularly emarginate (arcuately emarginate in *Namibdrilus* gen. nov.), the prosternal process present, plate-like (absent in *Namibdrilus* gen. nov.), the mesoventral process well developed (absent in *Namibdrilus* gen. nov.), the elytral surface relatively smooth and with apparent elytral striae and interstriae or at least their remnants (elytral surface rather rough and without any striae in *Namibdrilus* gen. nov.), seven abdominal ventrites with the first four connate (eight free ventrites in *Namibdrilus* gen. nov.), and the intercoxal process present on the first abdominal ventrite (absent in *Namibdrilus* gen. nov.). Members of clade S (*Illubaboria* Kundrata & Bocak, 2019; *Selasia*) usually have an apparent prosternal process, well-developed mesoventral process, seven abdominal ventrites, and at least a slightly developed intercoxal process on the first abdominal ventrite. The representatives of clade M (*Flabelloselasia* Kundrata & Bocak, 2017, *Kupeselasia* Kundrata & Bocak, 2017, *Lolosia* Kundrata & Bocak, 2017, *Microselasia* Kundrata & Bocak, 2017) are usually tiny forest species from the western and central Africa which differ from *Namibdrilus* gen. nov. in having a smooth glabrous pronotal disc often with distinct sublateral carinae (pronotum with relatively rough surface and no sublateral carinae in *Namibdrilus* gen. nov.) and the prosternum with a prosternal process forming a narrow plate (absent in *Namibdrilus* gen. nov.).

On the other hand, *Namibdrilus* gen. nov. shares many diagnostic characters with the Palaearctic genera included in clade D (*Drilorhinus*, *Drilus*, *Malacodrilus*, *Malacogaster*), including the frontoclypeal region produced forwards between antennae, the pronotum not distinctly transverse and without sublateral carinae, the posterior margin of pronotum shallowly and arcuately emarginate, the prosternum without a well-developed prosternal process, the scutellar shield apically subtruncate, the mesoventrite v-shaped and without a well-developed mesoventral process, the elytral apices not conjointly rounded and instead being divergent at internal margins, the elytral surface rough and without distinct striae, and the abdomen with eight free visible sternites. Based on the above-listed characters, we place this genus within clade D (*Drilus* clade) as the only Afrotropical member in this otherwise Palaearctic group. *Namibdrilus* gen. nov. differs from all other genera in the group by having much larger eyes, with their frontal separation 1.15 times eye diameter (eyes in the Palaearctic genera are small to medium-sized, with their frontal separation 1.60–3.00 times eye diameter; Fig. 2A–D; e.g., Kundrata et al. 2015a, Hoffmannova and Kundrata 2022), the frontoclypeal region strongly produced forwards, carinate and anteriorly widely rounded (if the frontoclypeal region in the Palaearctic genera is strongly produced then it is anteriorly narrowed and clearly emarginate; Fig. 2A–D; Packova et al. 2021), unidentate mandibles (always bidentate in the Palaearctic genera), relatively narrower prosternum, and the aedeagal median lobe without any subapical hook (Fig. 4E–G). Based on its divergent morphology and distribution in tropical Africa, we hypothesize that *Namibdrilus* gen. nov. may be a sister group to all remaining genera in clade D. Of course, without a strong phylogenetic hypothesis we cannot exclude the possibility that *Namibdrilus* gen. nov. either represents a more developed paedomorphic phenotype in one of the previously identified African clades (less likely) or it forms a separate clade on its own. All these hypotheses should be tested using a molecular approach in future research.

One of the most striking characters of *Namibdrilus* gen. nov. is the frontoclypeal region which is strongly produced forwards and is visible even from the ventral view of the head (Fig. 2D). Although Drilini have variously shaped frontoclypeal region from e.g., a very short and wide in *Selasia* spp. to a narrow and high in *Microselasia* spp., major modifications are known in genera of the *Drilus* clade. *Malacogasterruficollis* Dodero, 1925 from Libya is the only species of genus *Malacogaster* having the frontoclypeal region produced forwards and covering labrum; however, it is very wide, sloping downwards and broadly rounded apically (Hoffmannova and Kundrata 2022). On the other hand, the monotypic *Drilorhinus* from Iran (Fig. 5A) and several Mediterranean *Drilus* spp. have the frontoclypeal region strongly produced forwards and apically narrowed, with apex medially distinctly emarginate (Kovalev et al. 2019; Packova et al. 2021). None of these conditions is similar to the strongly produced frontoclypeal region of *Namibdrilus* gen. nov., which is oriented straight forwards and apically somewhat subtruncate and widely rounded (Fig. 2A–D).

In summary, the discovery of *Namibdrilus* gen. nov. is extremely important for our understanding of the diversity and evolution of Drilini and represents one of the most interesting findings regarding Drilini in recent decades. Recent increased research on Drilini has already led to a better understanding of their systematic placement (Kundrata and Bocak 2011), their phylogenetic relationships and diversity, including descriptions of several new genera (Kundrata et al. 2015a, Kundrata and Bocak 2017, 2019), or the discovery of the first Drilini from South East Asia (Kundrata and Sormova 2018). Based on morphology, *Namibdrilus* gen. nov. represents a connection between tropical Africa, the center of diversity of Drilini, with a solely Palaearctic group of genera with the highest degree of paedomorphosis-related morphological modifications (although this remains to be further tested using a molecular approach). Furthermore, its unique aedeagal morphology stresses the importance of male genitalia for the systematics of Drilini.

## Supplementary Material

XML Treatment for Namibdrilus

XML Treatment for Namibdrilus albertalleni

## ﻿Additional information

### Author ORCIDs

Robin Kundrata https://orcid.org/0000-0001-9397-1030

Gabriela Packova https://orcid.org/0000-0001-7949-619X

### Data availability

All of the data that support the findings of this study are available in the main text.
